# A Novel Frailty Index Can Predict the Short-Term Outcomes of Esophagectomy in Older Patients with Esophageal Cancer

**DOI:** 10.3390/curroncol31080349

**Published:** 2024-08-16

**Authors:** Thomas Boerner, Marisa Sewell, Amy L. Tin, Andrew J. Vickers, Caitlin Harrington-Baksh, Manjit S. Bains, Matthew J. Bott, Bernard J. Park, Smita Sihag, David R. Jones, Robert J. Downey, Armin Shahrokni, Daniela Molena

**Affiliations:** 1 Thoracic Service, Department of Surgery, Memorial Sloan Kettering Cancer Center, New York, NY 10065, USA; 2 Department of Epidemiology and Biostatistics, Memorial Sloan Kettering Cancer Center, New York, NY 10065, USA; 3 Department of Medicine, Memorial Sloan Kettering Cancer Center, New York, NY 10065, USA

**Keywords:** frailty, novel scoring system, older patients, esophagectomy

## Abstract

**Background:** Frailty, rather than age, is associated with postoperative morbidity and mortality. We sought to determine whether preoperative frailty as defined by a novel scoring system could predict the outcomes among older patients undergoing esophagectomy. **Methods:** We identified patients 65 years or older who underwent esophagectomy between 2011 and 2021 at our institution. Frailty was assessed using the MSK-FI, which consists of 1 component related to functional status and 10 medical comorbidities. We used a multivariable logistic regression model to test for the associations between frailty and short-term outcomes, with continuous frailty score as the predictor and additionally adjusted for age and Eastern Cooperative Oncology Group performance status. **Results:** In total, 447 patients were included in the analysis (median age of 71 years [interquartile range, 68–75]). Most of the patients underwent neoadjuvant treatment (81%), an Ivor Lewis esophagectomy (86%), and minimally invasive surgery (55%). A total of 22 patients (4.9%) died within 90 days of surgery, 144 (32%) had a major complication, 81 (19%) were readmitted, and 31 (7.2%) were discharged to a facility. Of the patients who died within 90 days, 19 had a major complication, yielding a failure-to-rescue rate of 13%. The risk of 30-day major complications (OR, 1.24 [95% CI, 1.09–1.41]; *p* = 0.001), readmissions (OR, 1.31 [95% CI, 1.13–1.52]; *p* < 0.001), and discharge to a facility (OR, 1.86 [95% CI, 1.49–2.37]; *p* < 0.001) increased with increasing frailty. Frailty and 90-day mortality were not associated. **Conclusions:** Frailty assessment during surgery decision-making can identify patients with a high risk of morbidity.

## 1. Introduction

Owing to demographic shifts and improvements in life expectancy, increasing numbers of older patients are being diagnosed with esophageal cancer. Physicians will be faced with the challenge of managing an aggressive disease in patients with multiple comorbidities and reduced physiological reserve. Adequate patient selection is essential to deliver effective treatment safely. There is accumulating evidence that older patients with locally advanced esophageal cancer can benefit from aggressive multimodal therapy, including perioperative chemotherapy and surgical resection [1]. Chronological age itself should not preclude patients from receiving potentially curative treatment; physicians should instead consider a patient’s functional status, comorbidities, and social support. Screening for frailty before the commencement of therapy and the use of comprehensive geriatric assessment are valuable tools for identifying patients with a higher risk of toxicity [2]. Popular measures of fitness in oncology include the Eastern Cooperative Oncology Group (ECOG) performance status or the Karnofsky performance scale. These measures were validated in younger populations and do not consider domains contributing to frailty such as medications, comorbidity, cognition [3], or interuser variability [4]. This suggests that these measures may have reduced validity in older populations, such as patients with esophageal cancer. Previous studies have shown that these measures are inferior to other frailty screening tools [5]. We previously described the development of the Memorial Sloan Kettering Frailty Index (MSK-FI), which comprises 10 comorbidities and 1 component related to functional assessment based on four patient-reported activities of daily living and one patient-reported instrumental activity of daily living [6]. Since our original publication, this novel frailty assessment tool has proven to be a valid instrument for predicting the short- and long-term postoperative outcomes in older patients with a variety of solid cancers [7,8].

In this study, we investigated whether frailty as defined by the MSK-FI can predict the short-term outcomes among older patients undergoing esophagectomy.

## 2. Materials and Methods

After approval from our institutional review board, we queried our prospectively maintained institutional database to identify all patients aged ≥65 years who underwent esophagectomy at our institution between January 2011 and March 2021. Of the initial 467 patients identified, 10 patients without an available preoperative adult health screening assessment (which was needed to calculate the functional component of the MSK-FI) and 10 patients without their ECOG performance status (which was a covariate included in our multivariable analysis) available were excluded.

The functional component of the MSK-FI was recorded by the clinical staff when the patients presented for their preoperative appointment, either in our clinic or by preoperative nursing staff. Comorbid conditions were recorded as discrete variables and were available in patient charts.

Our primary aim was to determine whether frailty as determined by the MSK-FI can predict short-term outcomes: mortality within 90 days of surgery, major complications within 30 days of surgery, readmissions within 30 days of discharge, and discharge to a facility (anywhere other than home, with a visiting nurse service). Death, which by definition would correspond to a grade 5 complication, was not considered a major complication within 30 days. Hence, a patient who died was classed as having a complication only if it was documented before the time of death.

We first visualized the unadjusted association in our cohort by plotting the probability of each outcome based on the MSK-FI score. We then created a multivariable logistic regression model for each outcome separately, with the continuous MSK-FI score as the predictor and with adjustment for continuous age at surgery and ECOG performance status. As we found no evidence of a nonlinear association between MSK-FI score and outcomes, MSK-FI score was included in the model as a linear variable. ECOG performance status ranges from 0 to 4, with higher values corresponding to a more limited self-care ability. In our cohort, no patients had an ECOG performance status >2, which corresponds to a status of “ambulatory and capable of all self-care but unable to carry out any work activities; up and about more than 50% of waking hours.” Therefore, ECOG performance status was included in the model as a categorical variable (0 vs. 1 vs. 2) for the outcomes of major complications within 30 days of surgery, readmissions within 30 days of discharge, and discharge to a facility. Because of the limited number of deaths within 90 days of surgery, ECOG performance status was included in this model as a binary variable (0 vs. 1 or 2) for this outcome. Patients who died in the hospital before discharge were not included in the analyses related to the outcomes of readmissions within 30 days of discharge and discharge to a facility.

A secondary aim was to determine the frequency of and the proportion of patients with “failure to rescue” within 90 days of surgery. This was defined as a death within 90 days of surgery subsequent to a major complication within 30 days of surgery. Moreover, we were interested in ascertaining the association between frailty, mortality within 90 days of surgery, and major complications within 30 days of surgery. To this end, we defined two other multivariable logistic regression models with mortality within 90 days of surgery as the outcome and with adjustment for age and ECOG performance status; the first model included the primary predictors of the MSK-FI score and major complications within 30 days of surgery, and the second model included both primary predictors, as well as an interaction term between the two primary predictors.

From our logistic regression models, we reported the odds ratios (ORs) and 95% confidence intervals (CIs). All the tests were 2-sided, and significance was set at *p* < 0.05. All the statistical analyses were conducted using STATA 15.0 (StataCorp, College Station, TX, USA) and R version 4.2.2 (R foundation for Statistical Computing, Vienna, Austria).

## 3. Results

### 3.1. The Cohort

In total, 447 patients met the study criteria and were included for analysis (median age of 71 years [interquartile range of 68–75]). Most patients underwent neoadjuvant treatment (81%), Ivor Lewis esophagectomy (86%), and minimally invasive surgery (55%). Most patients had adenocarcinoma (407/447, 88%) and clinical stage III disease (276/447, 62%) (Table 1). One patient did not have at least 90 days of follow-up and was excluded from all the analyses related to 90-day mortality; 15 patients died in the hospital before discharge and were therefore excluded from all analyses related to discharge to a facility and readmissions within 30 days of discharge.

### 3.2. Outcomes

In total, 22 patients (4.9% [95% CI, 3.2–7.5%]) died within 90 days of surgery, 144 patients (32% [95% CI, 28–37%]) had a major complication within 30 days of surgery, 81 patients (19% [95% CI, 15–23%]) were readmitted within 30 days of discharge, and 31 patients (7.2% [95% CI, 5.0–10%]) were discharged to a facility, including 25 patients sent to extended care rehabilitation, 5 sent to another hospital, and 1 sent to a nursing home. Further evaluation of the cause of death showed that of the 22 patients who died within 90 days of surgery, 2 died due to progression of disease. The remaining 20 patients did not die of the index disease: 19 had a major complication beforehand, and 1 died of myocardial infarction. Of the 446 patients with 90 days of follow-up, 143 had a major complication within 30 days of surgery (32%); of these, 19 died within 90 days of surgery, yielding a failure-to-rescue rate of 13% (95% CI, 8.4–20%). The median time between a major complication within 30 days of surgery and death within 90 days of surgery was 19 days (interquartile range, 1–47).

**Figure 1:**
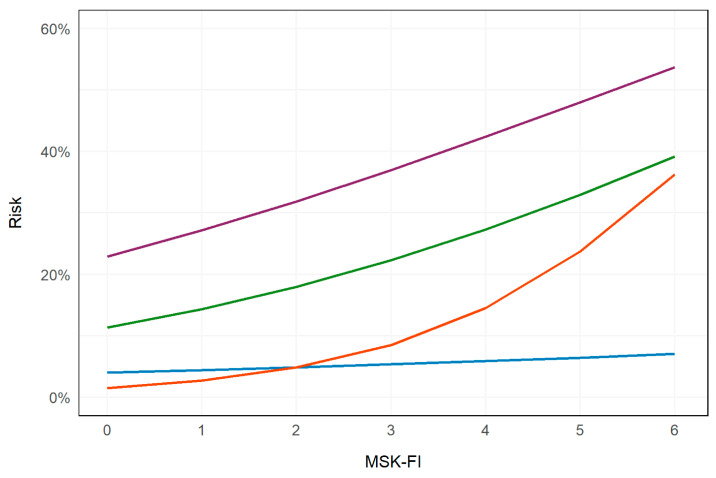
Risk of short-term outcomes based on Memorial Sloan Kettering Frailty Index (MSK-FI) score. Purple: major complication within 30 days of surgery; green: readmission within 30 days of discharge; orange: discharge to a facility; blue: mortality within 90 days of surgery. Three patients with an MSK-FI score >6 (one with an MSK-FI score of 7 and two with an MSK-FI score of 8) are omitted from the figure.

Figure 1 presents the probability of each short-term outcome based on MSK-FI score; Table 2 displays the association between MSK-FI score and short-term outcomes for the adjusted analyses. Risk of death within 90 days of surgery, a major complication within 30 days of surgery, readmission within 30 days of discharge, and discharge to facility increased with increasing frailty; however, the association between frailty and risk of death within 90 days of surgery was not statistically significant (*p* = 0.3) (Table 2).

Overall, the mortality within 90 days of surgery was higher for patients with a major complication within 30 days of surgery than for patients without a major complication within 30 days of surgery (13% vs. 1.0%, difference = 12% [95% CI, 6.1–18%]; *p* < 0.001). In our prespecified analysis of the association between frailty, mortality within 90 days of surgery, and major complications within 30 days of surgery, the inclusion of major complications within 30 days of surgery reduced the association between MSK-FI score and 90-day mortality (OR, 1.01 [95% CI, 0.76–1.32]; *p* > 0.9). Moreover, a major complication within 30 days of surgery was associated with a higher odds of subsequent death within 90 days of surgery compared with a lack of a major complication within 30 days of surgery (OR, 15.6 [95% CI, 5.13–67.8]; *p* < 0.001).

## 4. Discussion

The optimal management of older patients with esophageal cancer is of increasing importance in an aging society and for a disease with a peak incidence that has shifted beyond 70 years of age in recent years. The concept of frailty has emerged as an important predictor that is associated with a higher risk of death, postoperative complications, and healthcare expenditures after major surgery in older patients with cancer [9,10,11].

The impact of frailty on postoperative complications in patients who undergo esophagectomy is a matter of debate. Several retrospective studies have investigated the postoperative morbidity among older patients in the context of radical surgical therapy. Although some studies found no association between frailty and major postoperative complications [12,13], most of the available data suggest—as in the present study—that higher frailty among patients with esophageal cancer is associated with a higher risk of serious postoperative complications [14,15,16,17]. The negative findings in some of these studies might be explained by the inclusion of highly selected cohorts of “fit” patients, as many surgeons avoid overly invasive surgical procedures for high-risk patients. At our institution, patients are not routinely denied esophagectomy on the basis of stringent criteria, and we aim to treat all cases of locally advanced esophageal cancer aggressively with multimodality therapy. The definitive decision to forgo surgery and opt for less invasive therapy, such as definitive chemoradiation, is mainly based on the preference of the treating physician, lack of a surgical consultation, a decline in performance status, or the patient’s preference to avoid surgery.

The 30-day mortality (2.2%) and 90-day mortality (4.9%) in our cohort, which comprised patients aged ≥65 years, were comparable to the rates observed after esophagectomy among younger and healthier patients (2.4% and 4.5%, respectively) [18]. Therefore, older patients with esophageal cancer should not be precluded from aggressive surgical management on the basis of chronological age alone. This finding further adds to the evidence that a patient’s frailty status should dictate patient management. The low mortality in our series is likely attributable to the high volume of patients with esophageal cancer who are treated regularly at our center and the associated surgical skills and experience of the treating physicians [19]. In addition, major advances in perioperative care, with the implementation of enhanced recovery after surgery programs, minimally invasive surgical techniques, and prehabilitation programs, may have also contributed to these positive outcomes [20,21,22]. However, despite the low mortality rate, major complications still occurred in nearly one-third of patients (31%), which is similar to the rates reported by others [23,24]. More importantly, frailty as assessed by the MSK-FI was associated with a higher risk of developing a major complication within 30 days of surgery. In addition, major complications within 30 days of surgery were associated with a higher risk of death within 90 days of surgery.

Of the 137 patients (31%) who developed major complications within 30 days of surgery, 19 died within 90 days of surgery, yielding a failure-to-rescue rate of 14%. The failure-to-rescue rate is an evolving concept for measuring the quality of surgical care [25,26,27]. Only a few studies have investigated failure to rescue after esophagectomy. In a study by Abdelsattar et al. that used data from the American Nationwide Readmission Database, 7130 of 26,820 patients who underwent esophagectomy (26.6%) experienced a major complication postoperatively, of whom 1321 (18.5%) died during the index hospitalization. In contrast to our findings, in a study by Liou et al. that used data from the American College of Surgeons National Surgical Quality Improvement Program, the failure-to-rescue rate after esophagectomy was only 6% [28]. An older age (>75 years), African American race, an American Society of Anesthesiologists Physical Status of 4 to 5, and major complications were independent predictors of failure to rescue after esophagectomy. However, the majority of the patients included in that study were aged <65 years, and these patients had fewer preexisting comorbidities than those in our study, which may have contributed to the lower rate.

Frail patients have a lower functional reserve, a higher vulnerability to adverse outcomes, and a limited capacity to survive the stressors of a major complication [29]. Frailty as assessed by the MSK-FI was also associated with a higher risk of loss of autonomy and the inability to return home after esophagectomy, which is in line with previous reports [30,31]. This finding is highly relevant, as health-related quality of life and loss of autonomy are just as important or even more important than survival in an aging population [32].

Although many different tools for assessing frailty have been developed, no one tool has emerged as the reference standard. There is some evidence that performance measures, such as handgrip strength, can act as a marker of frailty for patients undergoing elective surgery [33]. Most clinical trials in oncology have used ECOG performance status or the Karnofsky performance scale to evaluate the functional fitness of patients for systemic therapy. However, both lack granularity, have wide interuser variability, and have questionable applicability to surgical interventions [3,4]. If only ECOG performance status had been used to assess physiologic fitness in the patients undergoing esophagectomy in our study, nearly all of the patients in our cohort would have been deemed to be clinically fit, as 96% had an ECOG performance status of ≤1. However, assessment via the MSK-FI displays a clear separation between patients, leading to better stratification by risk of major short-term adverse outcomes. That frailty as assessed by the MSK-FI remained associated with the short-term outcomes in our multivariable analysis after adjustment for ECOG performance status shows that our novel frailty score offers additional value to the established measures.

Another major benefit of the MSK-FI is that it can be easily implemented in routine clinical care, even in a busy surgical clinic, as it is mainly based on preexisting comorbidities, with one component of functional assessment that can be included as part of the routine medical assessment during clinic visits [6]. Implementation in a clinical setting would allow timely referrals for comprehensive geriatric assessment to identify the potential causes of frailty and would allow providers to tailor their therapeutic and supportive care interventions accordingly [34]. In addition, a more accurate assessment of surgical risk will help physicians and patients in shared patient–physician decision-making.

Definitive chemoradiation with close surveillance is often offered as an alternative treatment to older patients with esophageal cancer, even if their disease is at a curable stage, and surgery is offered in only a minority of cases [35,36,37,38]. However, such alternative treatment options have major drawbacks. Modern chemoradiation regimens result in complete tumor eradication in approximately 50% of cases of esophageal squamous cell carcinoma and 25% of cases of esophageal adenocarcinoma [39]. We recently reported that delayed esophagectomy for persistent or recurrent disease after definitive chemoradiation is associated with a higher odds of major complications, compared with regular trimodality therapy—a finding that should be taken into account during patient counseling.

Our study has several limitations. First, it was performed at a single high-volume institution with relatively low rates of complications and low mortality. Second, as in any surgical study, it only includes patients selected by surgeons to be suitably fit for surgery. Physicians tend to avoid performing major surgeries in patients with esophageal cancer with a high-risk perioperative profile and instead opt for definitive chemoradiation as the primary treatment. Patients who were not considered for surgery—because of physician or patient preference, the lack of a surgical consultation, or a decline in performance status—were not included in our analysis. It may be that with more formal frailty assessments, more patients with esophageal cancer would have undergone surgery during the study period instead of nonoperative treatment.

Our study also has several strengths. This is one of the largest studies to investigate the association between frailty and short-term outcomes among esophagectomy patients aged ≥65 years. As frailty was assessed retrospectively, perioperative care was not modified by the treating healthcare providers based on frailty status; therefore, the risk of performance bias is low.

The use of frailty assessment in surgical decision-making can identify patients with a high risk of morbidity. Frail patients should be counseled about the risks and benefits of treatment in a multidisciplinary setting, including experts in surgery, oncology, and geriatric medicine. Future research should focus on the clinical utility of incorporating frailty assessment into routine clinical care.

## Figures and Tables

**Table 1:** Patient characteristics (N = 447). Clinical stage based on the 8th edition of the American Joint Committee on Cancer staging manual.

Characteristic	Total
Age at surgery, years	71 (68–75)
Male sex	361 (81)
Race	
White	395 (88)
White Hispanic	4 (0.9)
Black	6 (1.3)
Asian	25 (5.6)
Unknown	17 (3.8)
Body mass index	27.7 (24.9–31.0)
American Society of Anesthesiologists Physical Status	
2	25 (5.6)
3	375 (84)
4	47 (11)
Memorial Sloan Kettering Frailty Index score	
0	73 (16)
1	125 (28)
2	118 (26)
3	57 (13)
4	38 (8.5)
5	19 (4.3)
≥6	17 (3.8)
Forced expiratory volume, L	2.9 (2.3–3.4)
Unknown forced expiratory volume	121
ECOG performance status	
0	218 (49)
1	210 (47)
2	19 (4.3)
Pulmonary comorbidities	52 (12)
Cardiac comorbidities	316 (71)
Diabetes comorbidities	104 (23)
Renal comorbidities	11 (2.5)
History of smoking cigarettes	
Current	27 (6.0)
Former	285 (64)
Never	135 (30)
Tumor type	
Adenocarcinoma	393 (88)
Squamous cell carcinoma	48 (11)
Other	6 (1.3)
Siewert classification	
1	191 (43%)
2	153 (34)
3	29 (6)
NA	74 (17)
Clinical stage	
1	57 (13)
2	57 (13)
3	276 (62)
4	55 (12)
NA	2 (0.1)
Neoadjuvant treatment	360 (81)
Chemoradiation	349 (78)
Type of operation	
Ivor Lewis esophagectomy	384 (86)
Three-hole esophagectomy	32 (7)
Transhiatal esophagectomy	16 (4)
Partial or complete gastrectomy and esophagectomy	5 (1)
Other	10 (2)
Minimally invasive surgical approach	248 (55)
Resection margin	
R0	423 (95%)
R+	24 (5%)

Data are medians (interquartile ranges) or no. (%).

**Table 2:** Estimates from multivariable logistic regression.

Outcome	No.	Event No.	OR	95% CI	*p*
Death within 90 days of surgery	446	22	1.14	0.87–1.47	0.3
Major complication within 30 days of surgery	447	144	1.24	1.09–1.41	0.001
Readmission within 30 days of discharge	432	81	1.31	1.13–1.52	<0.001
Discharge to a facility	432	31	1.86	1.49–2.37	<0.001

## Data Availability

The datasets presented in this article are not readily available because of privacy and legal restrictions. Requests to access the datasets should be directed to Daniela Molena MD.
